# Retracted: The Association between the Parents' Knowledge of Carbohydrate Counting and the Glycaemic Control of the Children with Type 1 Diabetes

**DOI:** 10.1155/2019/5958948

**Published:** 2019-01-09

**Authors:** International Journal of Pediatrics

At the request of the authors, the article titled “The Association between the Parents' Knowledge of Carbohydrate Counting and the Glycaemic Control of the Children with Type 1 Diabetes” [1] has been retracted. It was found that the data used for the analysis in this article contains duplication of several records, as the authors discovered that 41 out of sample size of 181 were duplicated, which made the number inaccurate. This duplication affected the analysis and the results presented in the article. The authors apologize for this inconvenience.
